# Supplementary material for: Lymphocyte Subpopulations Associated with Neutralizing Antibody Levels of SARS-CoV-2 for COVID-19 Vaccination
Source: Vaccines (Basel). 2022 Sep 17;10(9):1550. doi: 10.3390/vaccines10091550 (PMC9501134; doi:10.3390/vaccines10091550)
Supplement: Supplementary file 1 [file vaccines-10-01550-s001.zip › vaccines-1896852-supplementary.pdf]

Table S1. Details of clones and concentrations of antibodies used for flow cytometry study

Antibody	Panels	Clone	Fluorophores	Antibody (uL)/ 100uL blood
CD45	All panels, cytokine	2D1	APC-H7	2.5, 5*
CD3	I, III, IV, V VI, VII	UCHT1	V500	2.5
CD3	Cytokine	SK7	FITC	20*
CD4	I, VI, cytokine	L200	V450, BV421	2.5, 5*
CD4	III, V	SK3	V450	2.5
CD8	I, cytokine	SK1	PerCP-Cy5.5, APC	5, 5*
CD8	IV	RPA-T8	V450	2.5
CD19	I	SJ25C1	APC	2.5
CD19	II	HIB19	V500	2.5
CD16	I, cytokine	B73.1	PE-Cy7	2.5, 5*
CD16	VII	3G8	V450	2.5
CD56	I, cytokine	NCAM16.2	PE-Cy7	2.5, 5*
CD38	I, II	HB7	PE, PE-Cy7	5, 2.5
HLA-DR	I, V	L243	FITC	5
CD138	II	MI15	V450	2.5
IgM	II	G20-127	APC	10
IgD	II	IA6-2	FITC	5
CD21	II	B-ly4	PE	5
CD27	II	M-T271	PerCP-Cy5.5	2.5
CD45RA	III, IV, VI	HI100	FITC, PerCP-Cy5.5	5
CD45RO	III, IV, V	UCHL1	PerCP-Cy5.5	2.5
CCR3	III	5E8	PE	2.5
CCR5	III, IV	2D7/CCR5	PE-Cy7, PE	2.5, 5
CCR7	III, IV	3D12	Alexa 647	2.5
CCR6	IV	11A9	PE-Cy7	2.5
CD25	V	2A3	PE	5
CD127	V	HIL-7R-M21	Alexa 647	5
TCR α/β	VI	WT31	FITC	5
CD31	VI	WM59	PE	5
CD62L	VI	DREG-56	PE-Cy7	5
TCR $\gamma\delta$	VI	B1	APC	5
CD94	VII	HP-3D9	FITC	5
CD314 (NKG2D)	VII	1D11	PE	5
CD161	VII	HP-3G10	BB700	2.5
CD122	VII	Mik-β3	APC	5
TNF-α	Cytokine	MAb11	PE	5*
IFN-γ	Cytokine	4S.B3	PerCP-Cy5.5	5*
IL-2	Cytokine	MQ1-17H12	BV510	5*
IL-21	Cytokine	3A3-N2.1	PE	5*
TGF-β	Cytokine	TW4-2F8	PerCP-Cy5.5	5*
IL-17	Cytokine	N49-653	BV510	5*

* The quantities of antibodies used for cytokine assays; Vendor: BD Biosciences
